# Modification of bacterial microcompartments with target biomolecules *via* post-translational SpyTagging†

**DOI:** 10.1039/d3ma00071k

**Published:** 2023-06-12

**Authors:** David M Beal, Mingzhi Liang, Ian Brown, James D Budge, Emily R Burrows, Kevin Howland, Phoebe Lee, Sarah Martin, Andrew Morrell, Emi Nemoto-Smith, Joanne Roobol, Maria Stanley, C Mark Smales, Martin J Warren

**Affiliations:** a School of Biosciences, Division of Natural Sciences, University of Kent Canterbury UK d.m.beal@kent.ac.uk c.m.smales@kent.ac.uk; b GSK Stevenage UK; c Catapult: Cell and Gene Therapy London UK; d Charles River Cambridge UK; e National Institute for Bioprocessing Research and Training, Foster Avenue, Mount Merrion, Blackrock, Co Dublin A94 X099 Ireland; f Quadram Institute Bioscience Norwich UK warrenm@nbi.ac.uk; g Norwich Medical School, University of East Anglia Norwich UK

## Abstract

Bacterial microcompartments (BMCs) are proteinaceous organelle-like structures formed within bacteria, often encapsulating enzymes and cellular processes, in particular, allowing toxic intermediates to be shielded from the general cellular environment. Outside of their biological role they are of interest, through surface modification, as potential drug carriers and polyvalent antigen display scaffolds. Here we use a post-translational modification approach, using copper free click chemistry, to attach a SpyTag to a target protein molecule for attachment to a specific SpyCatcher modified BMC shell protein. We demonstrate that a post-translationally SpyTagged material can react with a SpyCatcher modified BMC and show its presence on the surface of BMCs, enabling future investigation of these structures as polyvalent antigen display scaffolds for vaccine development. This post-translational ‘click’ methodology overcomes the necessity to genetically encode the SpyTag, avoids any potential reduction in expression yield and expands the scope of SpyTag/SpyCatcher vaccine scaffolds to form peptide epitope vaccines and small molecule delivery agents.

## Abstract 2

A post-translational strategy for the production of SpyTagged molecules suitable for use in Spy based conjugations is described.

## Graphical abstract

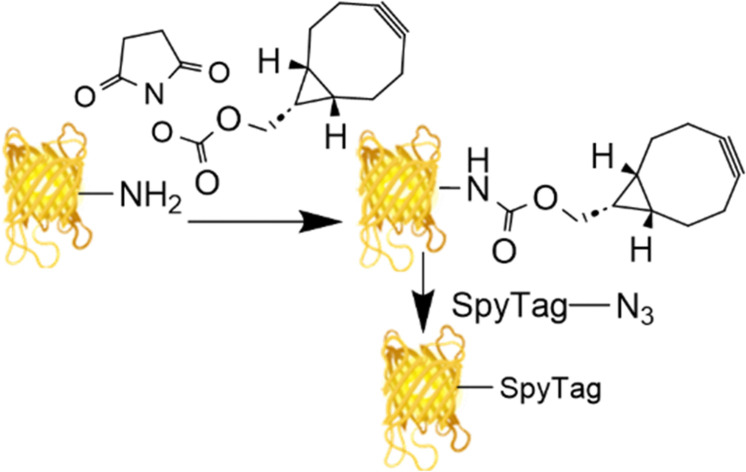

## Introduction

The ability to modify biological macromolecules and systems (*e.g.* proteins, DNA/RNA, cellular compartments and cells) with materials such as other proteins, fluorescent molecules, or small molecules (*e.g.* small molecule drugs) is increasingly important for many areas of research including targeted therapeutics such as antibody drug conjugates (ADCs),^1^ immunotherapies,^2,3^ vaccines^4^ and the fundamental investigation of cellular processes.^5^ There are many methodologies for conjugating proteins to different payloads including functionalisation of canonical^6^/non-canonical amino acids^7^ and incorporation of components by enzymatic modification (*e.g.* SnapTag,^8,9^ sortase^10^ and halotag^11^). Traditionally, despite giving rise to heterogeneous products, reacting canonical amino acids such as lysine and cysteine with modified maleimides, aldehydes and activated esters to incorporate functionality has been appealing due to the simplicity of the approach. Recent advances in site-specific unnatural amino acid incorporation have allowed for the generation of proteins that contain biorthogonal handles (azides and alkynes) suitable for targeted conjugation.^12^ Whilst the incorporation of such functionality gives the ability to generate a homogeneous product, these methods increase the complexity of the host system due to the incorporation of new tRNA synthetases and amino acids and can influence the yield and potentially the function of the target protein.^13^ Furthermore, such an approach results in a fixed system that requires additional new tRNA synthetases/amino acids for the incorporation of other functionality.

**Fig. 1:**
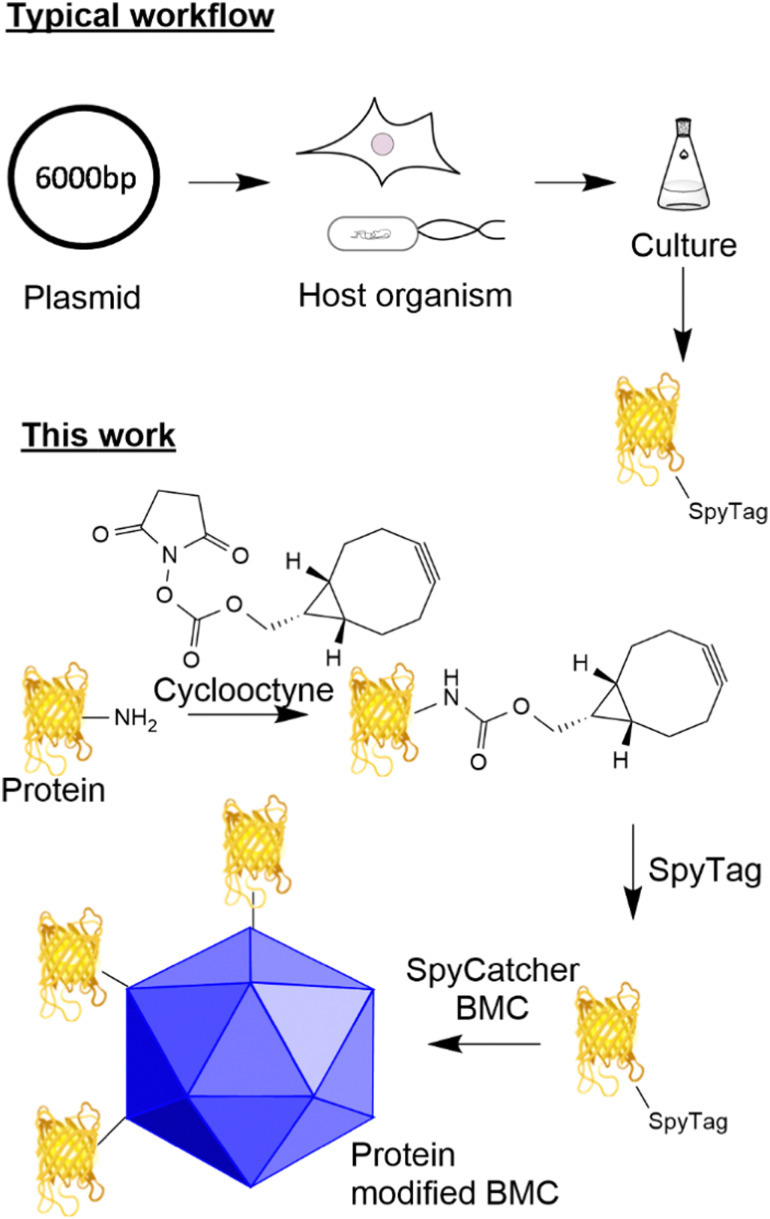
SpyTag peptides are typically incorporated through genetic encoding and recombinant protein expression. Previously, SpyTag modified proteins were produced recombinantly by the genetic modification of a host organism which produces the protein with the SpyTag attached. In this work, the post-translational modification of a protein, with a cyclooctyne, is used to modify the protein and allow the attachment of the SpyTag.

The utilisation of adaptor reagents^14^ that react with canonical/non-canonical amino acids to incorporate biorthogonal reactivity can prove useful to allow the flexible modification of cargo for subsequent modification by several different payloads. The ability to site-specifically target canonical amino acids enzymatically offers the advantage of site-specific modification of proteins without the requirement to engineer the host system further and offers the flexibility of modifying just the payload when a change of functionality is required. A well-established technique that allows specific site-directed targeting is the SpyTag/SpyCatcher pair which, when mixed, forms an isopeptide bond between an aspartic acid residue in the SpyTag and a lysine residue in the SpyCatcher.^15,16^ This method has significant advantages over other site-specific labelling approaches as it does not require enzyme catalysis to form the isopeptide bond and both reactive components can be encoded genetically in the protein sequence (see Fig. 1 for a typical workflow). This technology has found utility for the generation of vaccines^17,18^ and ADCs using an adapted approach.^19^

Bacterial microcompartments are organelle-like structures, which encapsulate functional enzymes and other proteins within a proteinaceous shell with a size ranging from 40 to 600 nm.^20^ The permeable shell is composed of three main types of shell proteins: hexameric BMC-H, trimeric/pseudomeric BMC-T and pentameric BMC-P.^21^ Recent reports have shown that these shell proteins, from a variety of BMC containing bacteria, can form empty BMCs (eBMCs).^22–26^ One of the most well studied BMCs is the Pdu bacterial microcompartment involved in 1,2-propanediol utilization in some enteric bacteria.^27^ Pdu eBMCs can be formed by heterologous expression of the *Citrobacter freundii* shell proteins, PduABB’JKN, in *E. coli*.^22^ It has recently been shown that PduAKN is the minimal shell protein requirements for forming Pdu eBMCs, and these minimal eBMCs are similar to their native form in both size and morphology (ref. 20). eBMCs can have the cargo targeted inside them, including enzymatic pathways, such that they encapsulate new processes for biotechnological processes,^28–30^ but they also have potential applications for drug delivery and polyvalent antigen display. Some of these potential applications require the ability to ‘decorate’ the exterior of eBMCs with cargo in a controlled manner.

Here, we describe the development of a SpyTag/SpyCatcher mediated conjugation methodology for the attachment of cargoes to eBMCs which allow the investigation of these new potential applications. We have observed that the incorporation of the SpyTag motif onto a protein can have detrimental effects on the yield of that protein from a given expression system (unpublished data). In parallel to the eBMC strategy, we describe the development of a method for the post-translational incorporation of a SpyTag onto a protein utilising lysine modification combined with strain promoted azide alkyne cycloaddition (SPAAC) chemistry (Fig. 1 this work). This post-translational modification method is exemplified using a model system, the fluorescent protein citrine, and a SpyCatcher modified Pdu eBMC. This methodology potentially allows access to SpyTagged proteins where genetic incorporation has proved difficult, or where genetic manipulation has not been possible.

## Results and discussion

### N_3_ SpyTag design and synthesis

SpyTag technology has evolved since its inception from an initial 1st generation SpyTag^16^ to a 2nd generation^31^ system and a 3rd generation^32^ system. There are many examples where these technologies have been utilised^17,18,33^ and there is evidence that there is some cross-reactivity between these systems.^34^ All three generations of peptides contain lysine residues, as well as an essential aspartic acid residue that is key for the reaction with SpyCatcher, but the lysine residues could be used for chemical modification. Modification of lysines using NHS ester-based amide bond forming chemistry has been used to incorporate fluorescent dyes^35^ but this adds multiple points of attachment so complicates subsequent modifications. Previously, a SpyTag maleimide^36^ has been generated for the modification of thiol-modified nanoparticles but this methodology is potentially complicated with regard to folding and disulfide isomerisation when proteins are utilised. To avoid these problems, the biorthogonal azide/alkyne reactive pair, core to the ‘click’ reactions CuAAC and SPAAC, can be incorporated onto the peptide backbone during solid phase peptide synthesis. Two peptides were designed using this strategy: the first representing the 1st generation SpyTag (SpyTag001) and the second the 3rd generation SpyTag (SpyTag003) (Fig. S1, ESI†).

The two peptides were synthesised using FMOC based solid phase peptide synthesis (SPPS, see Scheme S1, ESI†), using a TGT resin and HBTU as a coupling agent. The azide functionality was incorporated with a final coupling step on the N-terminal amine with 3-azidopropionic acid using the same conditions. The peptides, N_3_SpyTag001 and N_3_SpyTag003, were cleaved from the resin and purified by reverse phase HPLC purification (Fig. S2A and B, ESI†).

### Citrine modification – design, synthesis and conjugation

Citrine, a fluorescent protein, was used as a model protein to show the applicability of a two-step post-translational labelling strategy for the attachment of SpyTag to proteins. The fluorescence and structure of the protein, similar to GFP, offer advantages for the later workflow and attachment to BMCs as well as ease of production. As the SpyTag reagents are modified with an azide group, the citrine component needs to be modified with an alkyne functionality. In this instance a bicyclononyl cyclooctyne was used to take advantage of the SPAAC reaction occurring in the absence of copper. Citrine with an N-terminal his-tag, encoded within a pET14b plasmid (Fig. S3, ESI†), was produced by bacterial expression using an *E. coli* BL21* [DE3] pLysS strain. After cell lysis, the recombinant protein product was purified from the centrifuged-clarified supernatant by His-tag:Ni-NTA affinity chromatography and PD10 mediated buffer exchange.

**Fig. 2:**
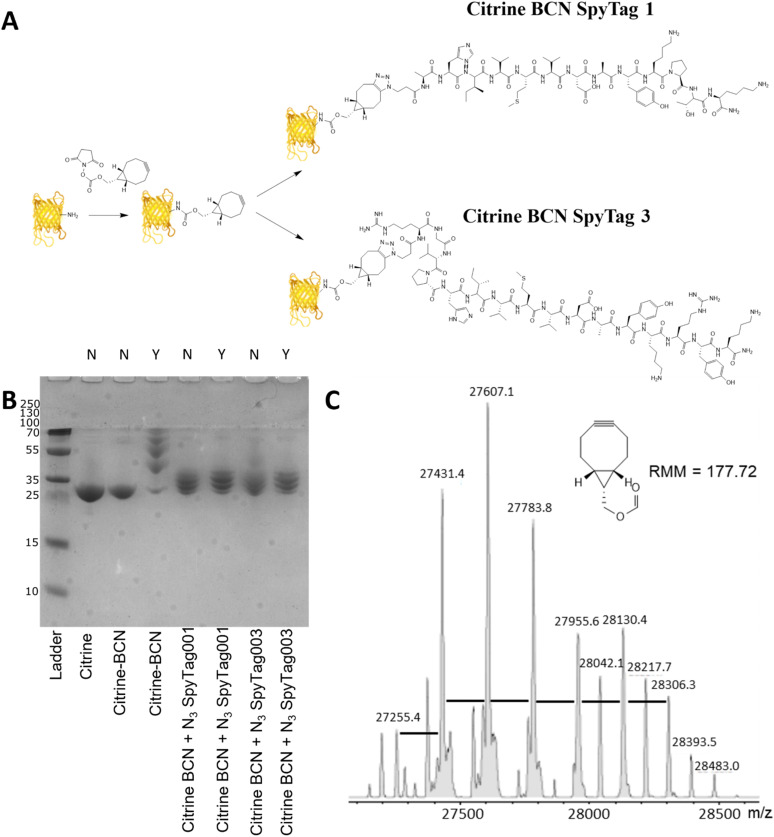
SpyTag modification of citrine through a two-step labelling strategy. (A) Reaction scheme showing the non-specific lysine modification of citrine using BCN-OSu to give a click competent protein. This was then modified using 1st and 3rd generation SpyTags, synthesised by solid phase synthesis, which was modified with an azide group. (B) SDS-PAGE analysis of the reaction of citrine with BCN-OSu and then subsequent reactions with N_3_ SpyTag001, 003 and also PEG-N_3_. Samples which have been treated with PEG-N_3_ are labelled as Y above the gel, and samples which do not have PEG-N_3_ are labelled as N. The gel was Coomassie stained to show changes in electrophoretic mobility of each sample. (C) Deconvoluted electrospray MS spectra of citrine-BCN showing consecutive additions of BCN (+176 Da) to citrine.

Citrine was activated for conjugation with the N_3_SpyTag molecules by the modification of lysine sidechains/N-terminal amino acids, using the activated bicyclononyl reagent ((1*R*,8*S*,9*s*)-Bicyclo[6.1.0]non-4-yn-9-ylmethyl-*N*-succinimidyl carbonate, BCN-OSu, Fig. 2A). Citrine (50 μM) was treated with 5 molar equivalents of 10 mM BCN-OSu (in DMSO) and then purified from excess reagents using a PD10 desalting column. Due to the small change in the molecular mass of citrine resulting from the modifications, no change in electrophoretic mobility was observed by SDS-PAGE (Fig. 2B, lane 2). Deconvoluted electrospray MS analysis of the BCN modification (Fig. 2C) shows a distribution of citrine modifications between 0 and 6 BCN molecules. To confirm the reactivity of the resulting BCN activated citrine (Citrine-BCN), the material was treated with 5k PEG N_3_ to give a species with a discernible band shift by SDS-PAGE (Fig. 2B, lane 3). This shows a trace of material with no band shift but with definite bands for the 1, 2, 3 and 4 additions.

To form citrine-SpyTag conjugates, the citrine-BCN was treated with 10 mM N_3_SpyTag001 and N_3_SpyTag003 and the reactions were incubated at 37 °C overnight, with excess peptide removed using a 5k molecular mass cut-off spin filter. SDS-PAGE analysis showed a smearing to a higher molecular mass upon modification by the N_3_SpyTag (Fig. 2B lanes 4 and 5), as expected. The citrine-SpyTag samples were also treated with 5k PEG-N_3_ to confirm no residual BCN remained (Fig. 2B lanes 6 and 7). The SpyTag001 sample (Fig. 2B lane 6) did show a trace of staining at higher molecular mass suggesting some free BCN. Mass spectrometry analysis of these reactions shows that for both Citrine-SpyTag001 and Citrine-SpyTag003 the desired products had formed but that there was also some fragmentation of the products during ionisation (Fig. S4 and S5, ESI†).

### eBMC construction and modification

Empty Pdu BMCs formed by co-expression of the three shell proteins PduA, PduK and PduN from the *Citrobacter freundii* propanediol utilisation system have recently been reported (ref. 28). In this study, the 3rd generation SpyCatcher eBMC (SCeBMC) was made by modifying PduK to give the SpyCatcher-PduK fusion protein, where the SpyCatcher was fused onto the C-terminus of PduK through genetic manipulation. Co-expression of SpyCatcher-PduK with the other proteins required to make the minimal eBMC, PduA and PduN resulted in the formation of SCeBMCs. The SCeBMCs generated were purified from the cellular debris and confirmation that the fusion of the SpyCatcher domain with PduK did not interfere with the formation of the BMC was observed by transmission electron microscopy (TEM) analysis (Fig. S6, ESI†).

**Fig. 3:**
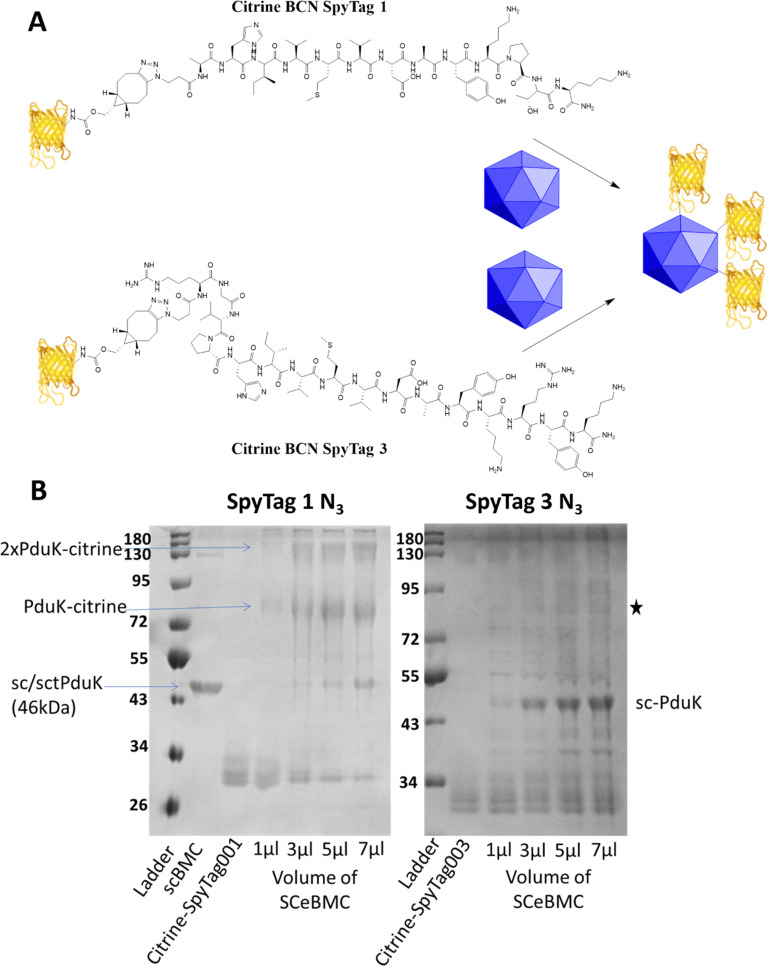
Post-translationally SpyTagged citrine (Citrine-SpyTag001/Citrine-SpyTag003) used to modify PduK-SpyCatcher fused empty Pdu bacterial microcompartments (SCeBMCs). (A) Reaction scheme showing the modification of BMCs using 1st and 3rd generation SpyTag conjugates through the PduK SpyCatcher. (B) SDS-PAGE analysis of the SpyCatcher BMC modification by SpyTag001 N_3_ and SpyTag003 N_3_. Shown are the SpyCatcher modified BMC (scBMC), the SpyTag modified citrine (st1citrine) and then 3 μL of SpyTag citrine treated with increasing amounts of scBMCs.

Conjugation of the SCeBMCs with the Citrine-SpyTag001/Citrine-SpyTag003 was achieved by mixing the purified SCeBMC (1 mg mL^−1^) with an excess of the Citrine-SpyTag001/Citrine-SpyTag003. To determine the saturation of the SCeBMC, a range of volumes (1, 3, 5 and 7 μL) at 1 mg mL^−1^ were reacted with a fixed volume (3 μL) of Citrine-SpyTag001 (∼280 μM) and Citrine-SpyTag003 (∼100 μM). SDS-PAGE analysis of the reactions showed that the SCeBMC reacted with Citrine-SpyTag001 to generate higher molecular mass products at 75 kDa and higher modifications due to the presence of multiply modified citrine (130 kDa/180 kDa). Interestingly, there was limited evidence of the reaction between Citrine-SpyTag003 and the SCeBMC despite having the correct 3rd generation SpyCatcher and 3rd generation SpyTag pair. Increasing the ratio of SCeBMC to Citrine-SpyTag003 did start to show evidence of a new product formed, marked with a star on Fig. 3B. By increasing the ratio of SCeBMC to Citrine-SpyTag001, saturation was observed at about 5–7 μl where the SpyCatcher-PduK fusion protein (75 kDa) becomes visible. Analysis of the Citrine-SpyTag001 bands (30 kDa) in the gel showed that as the SCeBMC increases the presence of SpyTagged modified citrine samples dissipated leaving only unmodified citrine left in the 5–7 μL SCeBMC samples.

**Fig. 4:**
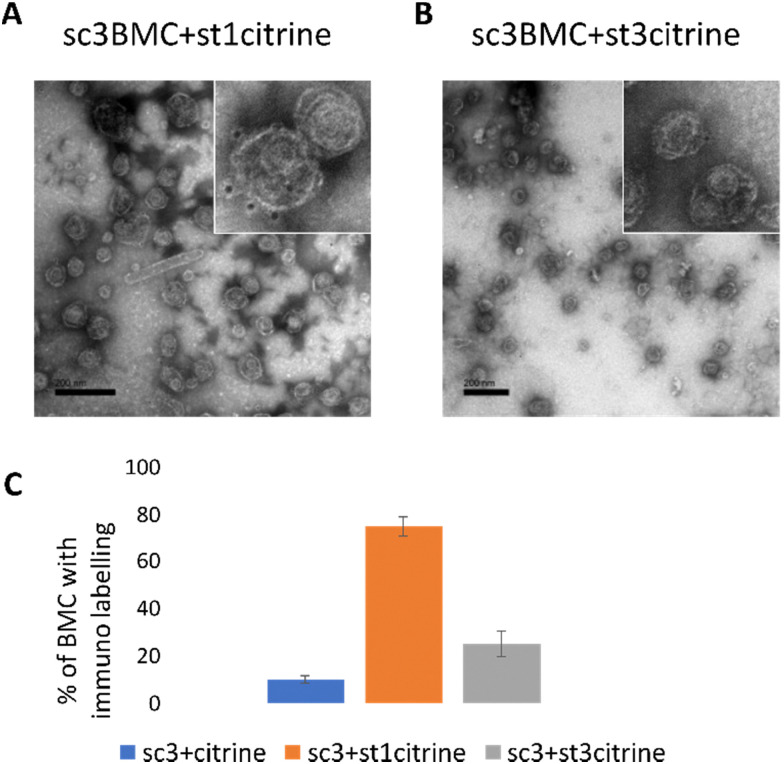
TEM imaging of SpyCatcher BMCs modified with SpyTag Citrine by immunogold labelling. (A) SpyCatcher BMCs modified with the Citrine SpyTag1 protein. (B) SpyCatcher BMCs modified with the Citrine SpyTag3 protein. (C) Quantification of the number of citrine molecules on the BMC surface.

To further confirm the conjugation of the SCeBMC to Citrine-SpyTag001 and explore the SDS-PAGE data associated with the Citrine-SpyTag003 reaction, samples were fixed on TEM grids and immunolabelled with anti-GFP antibodies (that recognise citrine) before being treated with gold nanoparticle conjugated secondary antibodies. Fig. 4A shows the presence of dark spots (gold nanoparticles) on the outside of the SCeBMC when treated with the Citrine-SpyTag001, confirming the attachment of citrine to the surface of the SCeBMC. Imaging of the Citrine-SpyTag003 modified sample shows some modifications of the SCeBMC with citrine (Fig. 4B), in agreement with the small amount of labelling observed by the SDS PAGE analysis. Quantification of the degree of SCeBMC labelling (Fig. 4C) by counting the number of gold nanoparticles associated with 150 SCeBMC particles revealed that as expected with Citrine-SpyTag001 there was a significant enhancement over the SCeBMC treated with unmodified citrine. The Citrine-SpyTag003 sample exhibited a much lower level of labelling compared to the Citrine-SpyTag001 sample (Fig. 4B and C) but did show a significant amount of labelling over the unmodified citrine control, suggesting that conjugation was successful but is apparently less efficient than in the Citrine-SpyTag001 reaction.

## Materials and methods

All chemicals were purchased from Merck Life Science UK Limited, Gillingham, Dorset, unless otherwise stated.

### Solid phase peptide synthesis (SPPS) of N_3_SpyTag001 and N_3_SpyTag003

Peptides were synthesised using a Shimadzu PSSM-8 Multiple Peptide Synthesiser using the fmoc strategy at 10 μM scale. Standard side chain protecting groups were utilised. The peptides were assembled on a pre-loaded TGT resin (Novabiochem) using HBTU mediated coupling and an 8-fold excess of the amino acid derivatives. Briefly, the fmoc group was removed by two 5 minute treatments with 20% piperidine in DMF followed by 5 washes with DMF. The amino acid (8 equiv.) was dissolved in DMF, mixed with 0.5 M HOBt/0.5 M HBTU in DMF (8 equiv.) and 1.0 M DIEA in DMF (16 equiv.) and added to the resin. The reaction was mixed by nitrogen bubbling for 45 minutes after which the resin was washed 5 times with DMF. This process was repeated until the required sequence was assembled. The azide group was incorporated by the addition of 3-aziodopropionic acid (Tebu-bio, Peterborough, Cambridgeshire), using the same coupling procedure as the standard amino acids.

The peptides were released from the resin and side chain deprotected by a single treatment with 1 mL of 94% TFA, 2.5% H_2_O, 2.5% EDT, and 1% TIS for 2 hours. The peptide was isolated by precipitation in ice cold diethyl-ether. The precipitate was washed 2 times with ice cold diethyl-ether to remove excess TFA/scavengers, dissolved in water and freeze dried.

The crude peptide was analysed by on-line reverse-phase LC-MS on a 150 × 2.1 mm Phenomenex Aeris Widepore 3.6 μm C18 300 Å column using an Agilent 1100 LC System coupled to a Bruker micrOTOF-Q II mass spectrometer using a H_2_O, Acetonitrile, 0.05% TFA gradient. The eluant was monitored at 214 nm and then directed into the electrospray source operating in positive ion mode, at 4.5 kV and the mass spectra were recorded from 50 to 3000 *m*/*z*. Data were analysed using Bruker's Compass Data Analysis software. Where necessary, the peptide was purified by semi-preparative reverse-phase HPLC on a 250 × 4.6 mm Vydac 5 μm C18 300 Å column with an Agilent 1100 LC system using a H_2_O, Acetonitrile, 0.1% TFA gradient. The final product was characterised by on-line reverse-phase LC-MS as for the crude peptide.

### Citrine production

A pET14b plasmid with the gene encoding citrine ligated between the *NdeI* and *SpeI* restriction sites (Fig. S3, ESI†) was used to produce citrine with a C-terminal HIS tag. The citrine encoded plasmid was transformed into the BL21* [DE3] pLysS strain of *E. coli* (supplier) by heat shock at 42 °C for 60 seconds. Transformants were selected by growth on LB agar plates containing 100 μg mL^−1^ ampicillin (Merck Life Science UK Limited, Gillingham, Dorset) and 35 μg mL^−1^ chloramphenicol (Merck Life Science UK Limited, Gillingham, Dorset). Individual transformed colonies were used to inoculate 10 mL of LB containing 100 μg mL^−1^ ampicillin and then grown overnight at 37 °C. The resulting cultures were used to inoculate 1 L of LB containing 100 μg mL^−1^ ampicillin and 35 μg mL^−1^ chloramphenicol. The cultures were grown to an OD_600_ of 0.6 before gene expression was induced by the addition of 400 μM isopropyl β-D-thiogalactopyranoside (IPTG) (Melford Laboratories, Ipswich, Suffolk) and incubated overnight at 19 °C. The cells were harvested by centrifugation at 4000 rpm for 10 mins before resuspension in 20 mM Tris-HCl, pH 8.0, containing 500 mM NaCl and 10 mM imidazole (Merck Life Science UK Limited, Gillingham, Dorset). The cell suspension containing citrine, yellow coloured, was lysed by sonication (6 min, 30 sec on/30 sec off at 55% amplitude). The resulting lysate was clarified by centrifugation at 18 000 rpm for 30 min at 4 °C. The resulting lysate was applied to a Ni-NTA column, equilibrated with 20 mM Tris-HCl, 500 mM NaCl, and 5 mM imidazole. The column was washed with 20 mM Tris-HCl, 500 mM NaCl, and 10 mM imidazole before washing with the same buffer with increasing amounts of imidazole (50 and 100 mM). The bound proteins were then eluted with 20 mM Tris-HCl, 500 mM NaCl, and 400 mM imidazole. The combined fractions were buffer exchanged into 20 mM Tris-HCl and 100 mM NaCl, pH 8, using a PD10 column (Cytvia Life Sciences, Sheffield, UK).

### Citrine-BCN synthesis and analysis

Citrine was buffer exchanged into 25 mM phosphate, pH 7.4, using a PD10 column. The resulting solution was diluted to 50 μM and then treated with 5eq BCN-OSu (Merck Life Science UK Limited, Gillingham, Dorset) (10 mM, DMSO (Merck Life Science UK Limited, Gillingham, Dorset) – stock prepared freshly before each conjugation). The reaction was incubated at 37 °C for 1 hour. The Citrine-BCN was purified from excess BCN-OSu and succinate by PD10 purification.

Aliquots (10 μL) of Citrine-BCN were treated with 1 μL of 10 mM 5k-PEG N_3_ (Merck Life Science UK Limited, Gillingham, Dorset) and incubated at 37 °C for 1 hour. Samples +/− PEG N_3_ were treated with a 4 × SDS-PAGE loading buffer + 5% 2-mercaptoethanol and heated to 95 °C for 5 min. The samples were then analysed using Tris-Tricine SDS-PAGE run at 180 V for 2 hours. The gel was stained with SafeBlue stain (NBS Biologicals, Huntingdon, Cambridgeshire).

Electrospray mass spectra analysis of the citrine BCN samples was performed using a Bruker micrOTOF-Q II mass spectrometer. A 200 pmole aliquot of each sample was desalted on-line by reverse-phase HPLC on a Phenomenex Jupiter C4 column (5 μm, 300 Å, 2.0 mm × 50 mm) running on an Agilent 1100 HPLC system at a flow rate of 0.2 mL min^−1^ using a short water, acetonitrile, 0.05% trifluoroacetic acid gradient. The eluent was monitored at 280 and 214 nm and then directed into the electrospray source, operating in positive ion mode, at 4.5 kV and the mass spectra were recorded from 500 to 3000 *m*/*z*. Data were analysed and deconvoluted to give uncharged protein masses with Bruker's Compass Data Analysis software.

### Citrine-SpyTag synthesis

Citrine-BCN (15 μM) was treated with 10 mM SpyTagN_3_ in DMSO (10 equiv. 150 μM). The reaction was incubated at 37 °C overnight. The resulting SpyTag conjugate was purified and concentrated by a series of dilution/concentrations using an Amicon 5 k MWCO spin filter (Merck Life Science UK Limited, Gillingham, Dorset). Aliquots were treated with the 4 × SDS-PAGE loading buffer + 5% 2-mercaptoethanol and heated to 95 °C for 5 mins. The samples were then analysed using Tris-Tricine SDS-PAGE run at 180 V for 2 hours. The gel was stained with SafeBlue stain. The electrospray analysis of the Citrine-SpyTag samples was carried out using the same conditions as for the Citrine-BCN samples.

### BMC preparation/purification^37^

Minimal Pdu BMCs can be formed by expressing three shell proteins PduA, PduK and PduN from *Citrobacter freundii* (submitted paper from Matt Lee). To add the SpyCatcher to the BMC, the *PduK* gene was fused with a 5′-3rd generation SpyCatcher sequence in a pET3a vector and the new vector can be used to express the fusion protein of SpyCatcher-PduK (sc-PduK). The modified BMC with a SpyCatcher was produced in *E. coli* BL21 (DE3) by co-expression of PduA, sc-PduK and PduN. Recombinant BMCs were extracted from *E. coli* and purified as described in the study by Liang *et al.*, 2017.^34^

### BMC modification

1, 3, 5 and 7 μL of purified SCeBMCs (1 mg mL^−1^) were mixed with 3 μl of Citrine-SpyTag001 (8.4 mg mL^−1^)/Citrine-SpyTag003 (3.05 mg mL^−1^) and incubated at 18 °C overnight. Excess citrine-SpyTag was removed by sedimentation of the modified eBMC by centrifugation (5 minutes, 13 000 rpm). The pellet was resuspended in the buffer at the concentration mentioned in the study by Liang *et al.*, 2017.^34^

### TEM analysis

Purified BMCs were loaded onto 400 mesh formvar and carbon coated gold grids followed by fixation with 2.5% glutaraldehyde in a 100 mM sodium cacodylate buffer, pH 7.2. The grids were equilibrated in one drop of TBST (20 mM Tris–HCl pH 7.4, 500 mM NaCl, 0.05% Tween 20, and 0.1% BSA) followed by blocking in 2% BSA in TBST for 30 minutes. The grids were then transferred into a dilution (1 : 100) of the primary antibody (rabbit anti-GFP polyclonal antibody, Thermofisher) and incubated for 1 hour at RT. The grids were washed with 6 drops of TBST (2 minutes per drop) before being placed into a drop of secondary antibody (goat anti-rabbit IgG 5 nm gold (Agar Scientific 1 : 50)) for 30 minutes. The grids were then washed as before with 6 drops of TBST followed by 6 drops of Milli-Q water. The grids were air dried before negative staining in 2% aqueous Uranyl acetate.

Samples were observed using a Jeol 1230 Transmission electron microscope at an accelerating voltage of 80 kV equipped with a Gatan One View digital camera.

## Conclusions

In conclusion, we have developed a post-translational modification strategy to incorporate the SpyTag peptide onto proteins without genetic engineering of their protein sequence. This two-step labelling strategy potentially allows any protein, independent of its origin, to be SpyTagged, although as with any methodology some protein specific optimisation is likely to be required. We suggest that the described labelling strategy may have widespread application providing an additional approach beyond genetic encoding to access the numerous platforms available to SpyCatcher technology, particularly for vaccine development.^17,18,38^

Future development work will focus on the utilisation of SpyTag-N_3_ for conjugation to small molecules, DNA and peptides using bioorthogonal technologies (CuAAC/SPAAC and Staudinger ligation). This has potential for expanding the scope of SpyTag/SpyCatcher vaccine scaffolds to form peptide epitope vaccines^4^ and small molecule vaccines^39–41^ but also difficult to express proteins. The successful modification of the surface a bacterial microcompartment allows for decoration with any cargo and will be invaluable for its investigation as a polyvalent antigen display scaffold.

## Author contributions

DMB and ML were responsible for the conceptualization, investigation, data analysis and writing – original draft and review and editing. CMS and MJW were responsible for the funding acquisition, conceptualization and writing – review and editing. IB, JDB, ERB, KH, PL, SM, AM, ENS, JR and MS were responsible for the investigation.

## Conflicts of interest

The authors confirm that there are no conflicts to declare.

## Supplementary Material

MA-004-D3MA00071K-s001
