# Supplementary material for: Modification of bacterial microcompartments with target biomolecules via post-translational SpyTagging
Source: Mater Adv. 2023 Jun 12;4(14):2963–70. doi: 10.1039/d3ma00071k (PMC10350929; doi:10.1039/d3ma00071k)

Electronic Supplementary Material (ESI) for Materials Advances.
This journal is © The Royal Society of Chemistry 2023

Modification of Bacterial Microcompartments by Target Biomolecules via Post-Translational SpyTagging.

David M Beal[a*†], Mingzhi Liang[a*], Ian Brown[a], James D Budge[a], Emily R Burrows[b], Kevin Howland[a], Phoebe Lee[a], Sarah Martin[c], Andrew Morrell[a], Emi Nemoto-Smith[d], Joanne Roobol[a], Maria Stanley[a], C Mark Smales[ae†], Martin J Warren[afe†]

Supplementary

Figure 1 – Scheme showing the azido SpyTag 1 N_3 and SpyTag 3 N_3 synthesised by SPPS.

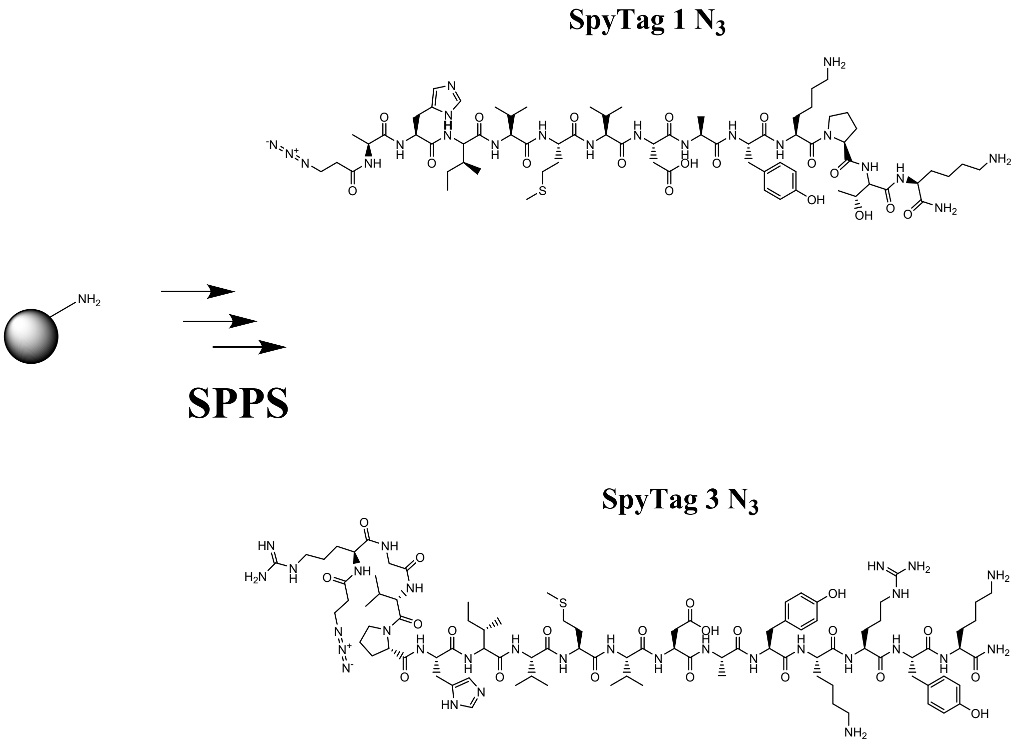

SpyTag 1 N_3 amino acid sequence

N_3 - AHIVMVDAYKPTK

SpyTag 3 N_3 amino acid sequence

N_3 - RGVPHIVMVDAYKRYK

Figure 2 – LCMS analysis of SpyTag 1 N_3 (A) and SpyTag 3 N_3 (B)

A

SpyTag 1 N_3 RMM = 1568.86 Da

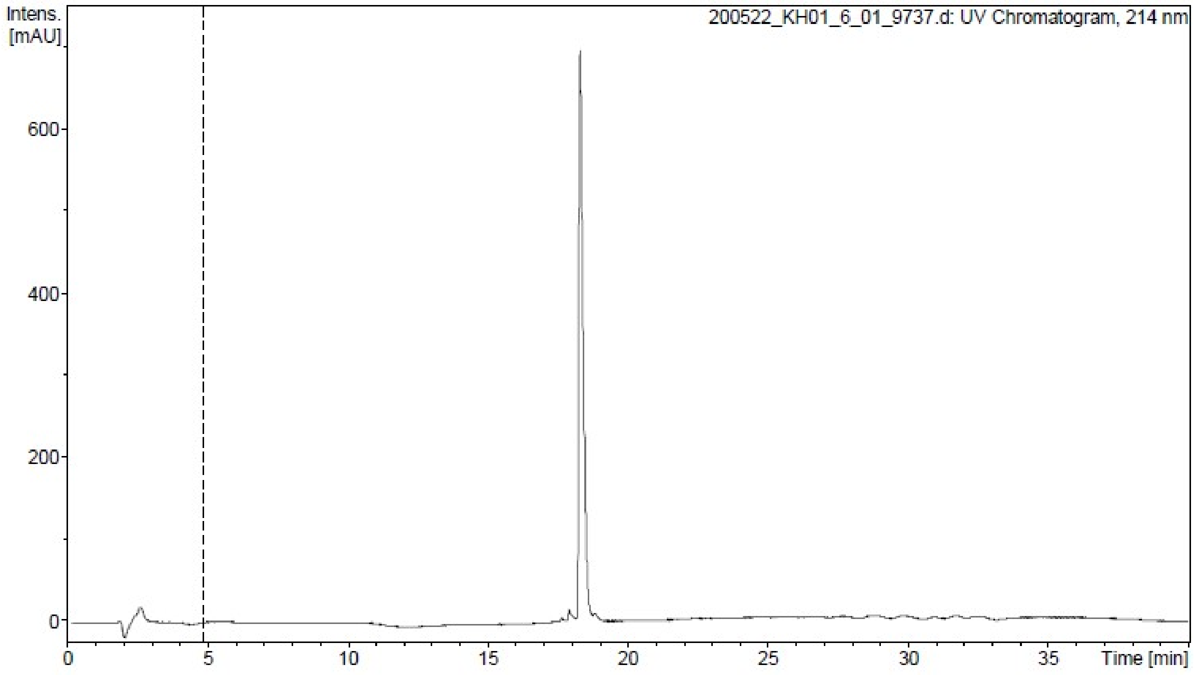

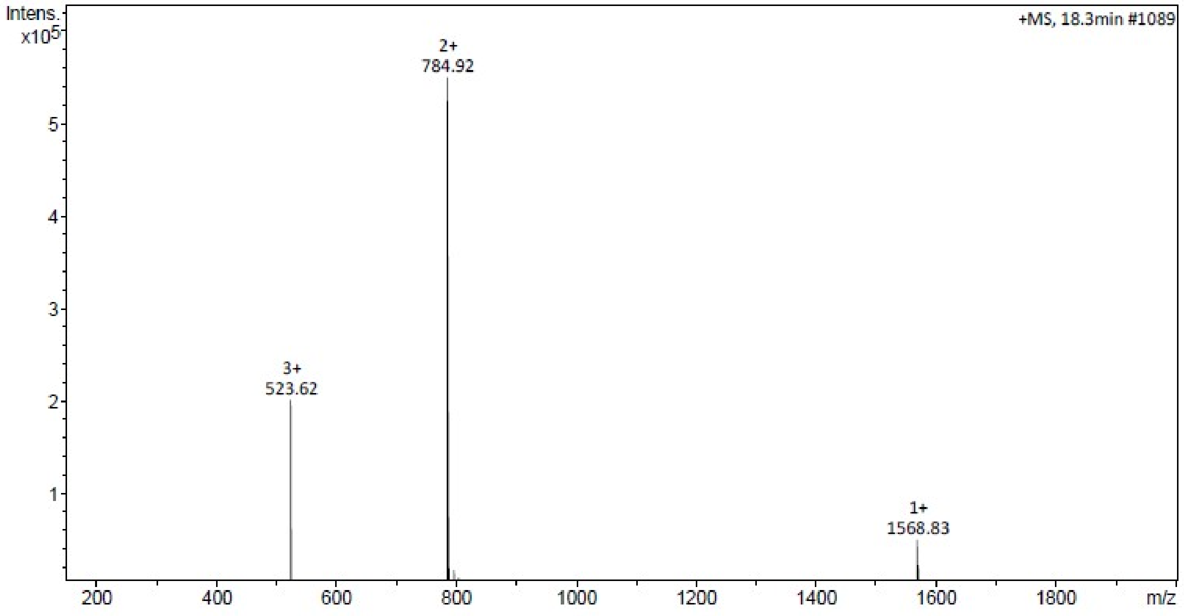

B

SpyTag 3 N_3 RMM = 2028.42 Da

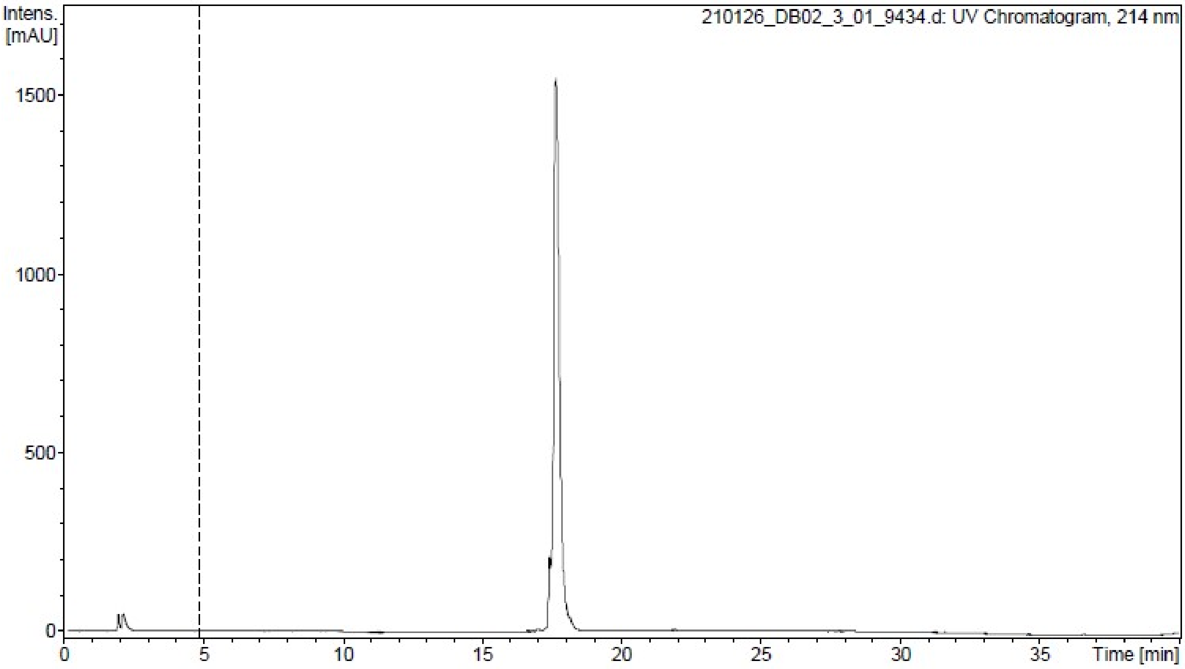

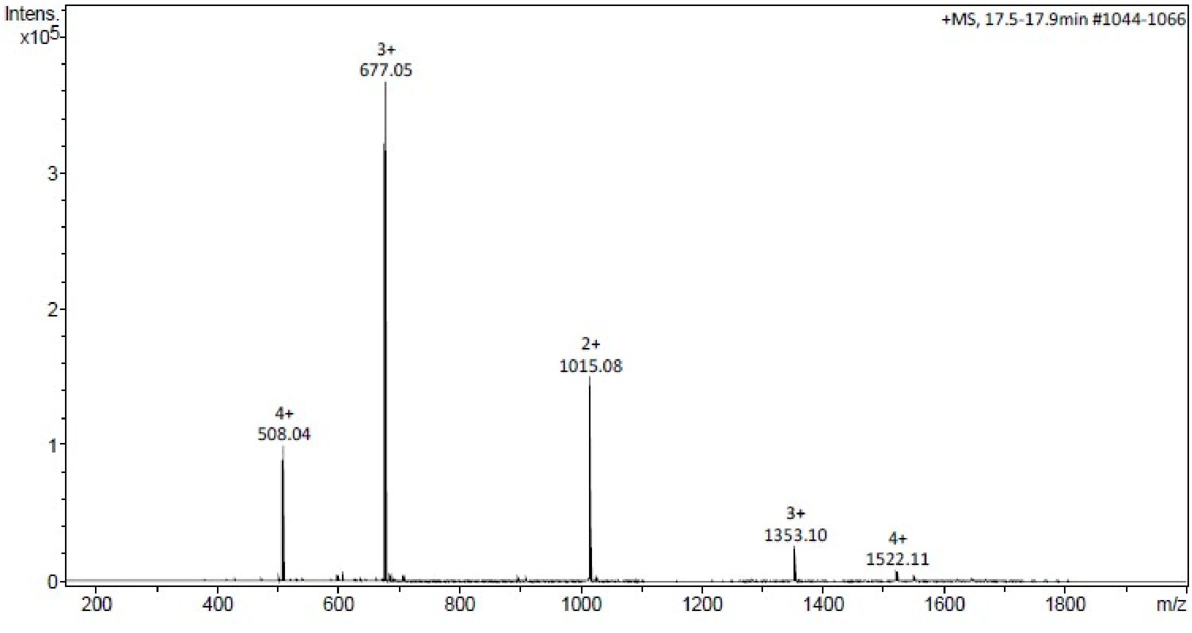

Figure 3. His tagged Citrine - plasmid map (A) and protein sequence (B)

A

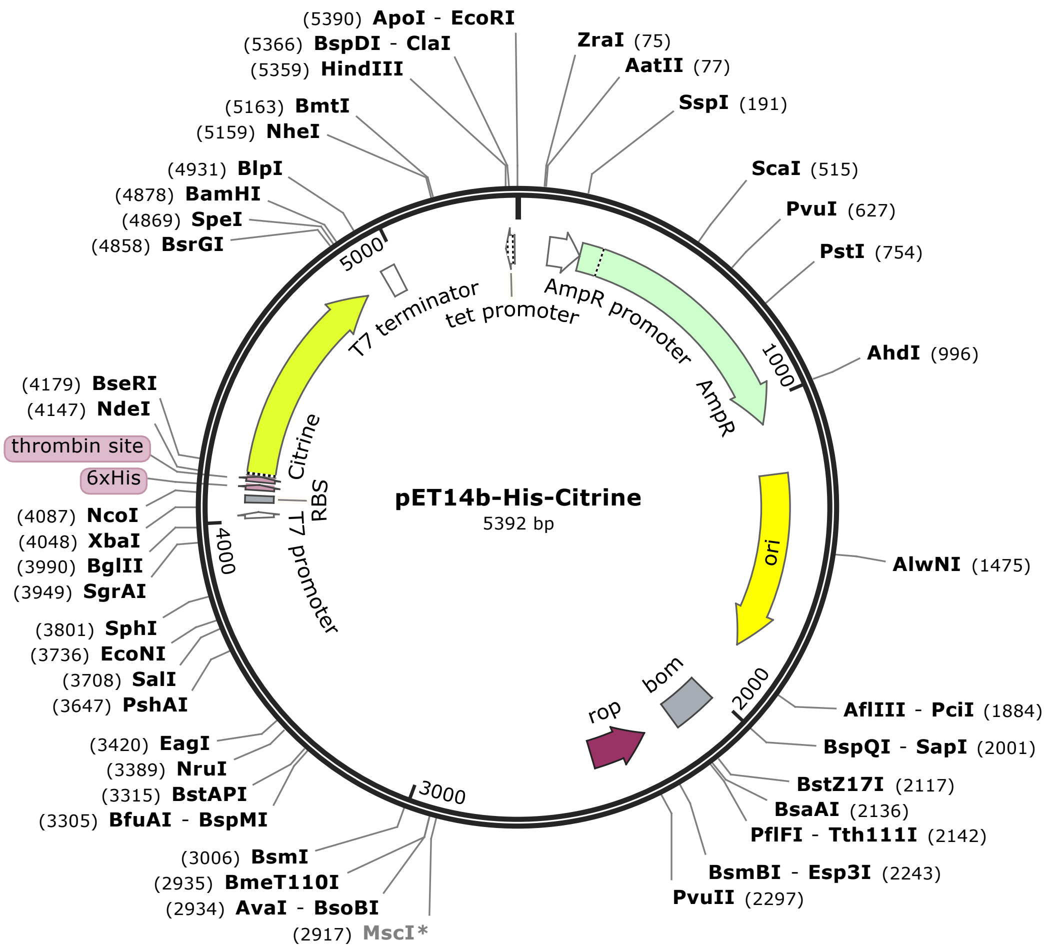

B. Citrine amino acid sequence

MGSSHHHHHHSSGLVPRGSHMVSKGEELFTGVVPILVELDGDVNGHKFSVSGEGEGDATYGKLTLKFICTTGKLPV

PWPTLVTTFGYGLMCFARYPDHMKQHDFFKSAMPEGYVQERTIFFKDDGNYKTRAEVKFEGDTLVNRIELKGIDF

KEDGNILGHKLEYNYNSHNVYIMADKQKNGIKVNFKIRHNIEDGSVQLADHYQQNTPIGDGPVLLPDNHYLSYQS

ALSKDPNEKRDHMVLLEFVTAAGITLGMDELYK

Figure 4 ES MS analysis of the Citrine BCN SpyTag1 N_3 conjugate.

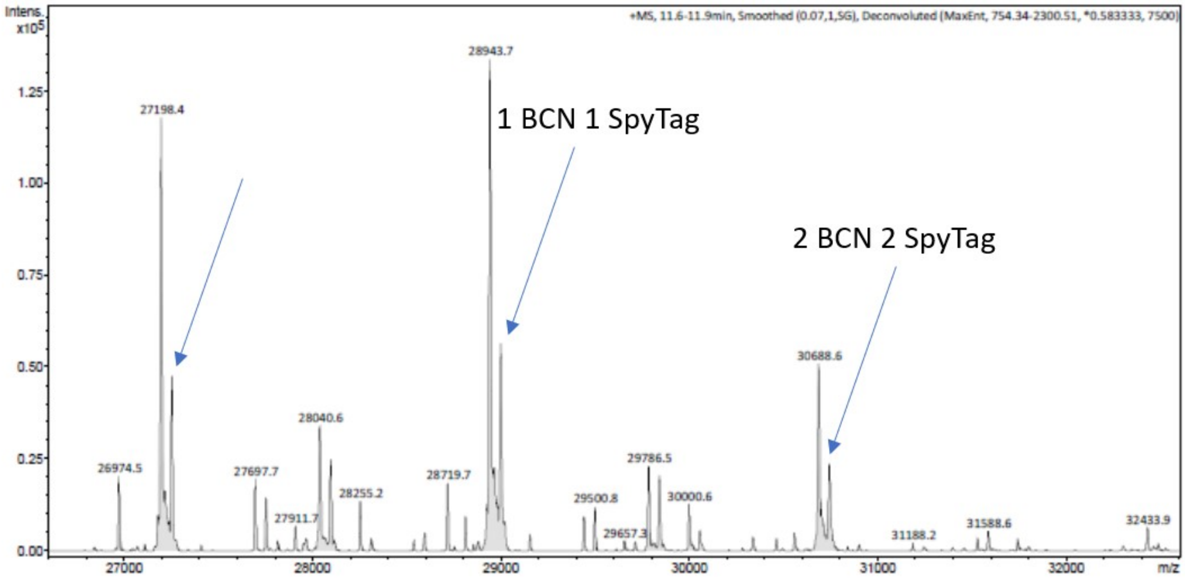

Figure 5 ES MS analysis of the Citrine BCN SpyTag1 N_3 conjugate.

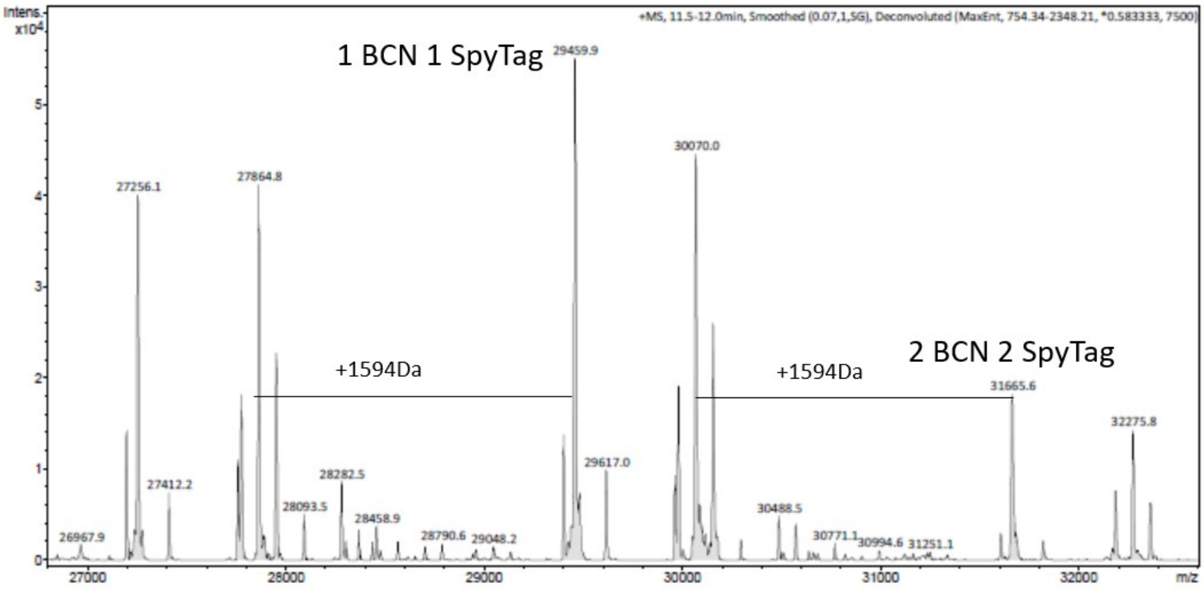

Figure 6 Electron micrographs of purified BMC. A) eBMC formed by three shell proteins. B) sc-eBMC form by three shell proteins with spycatcher sequence fused in one of the shell proteins, PduK.

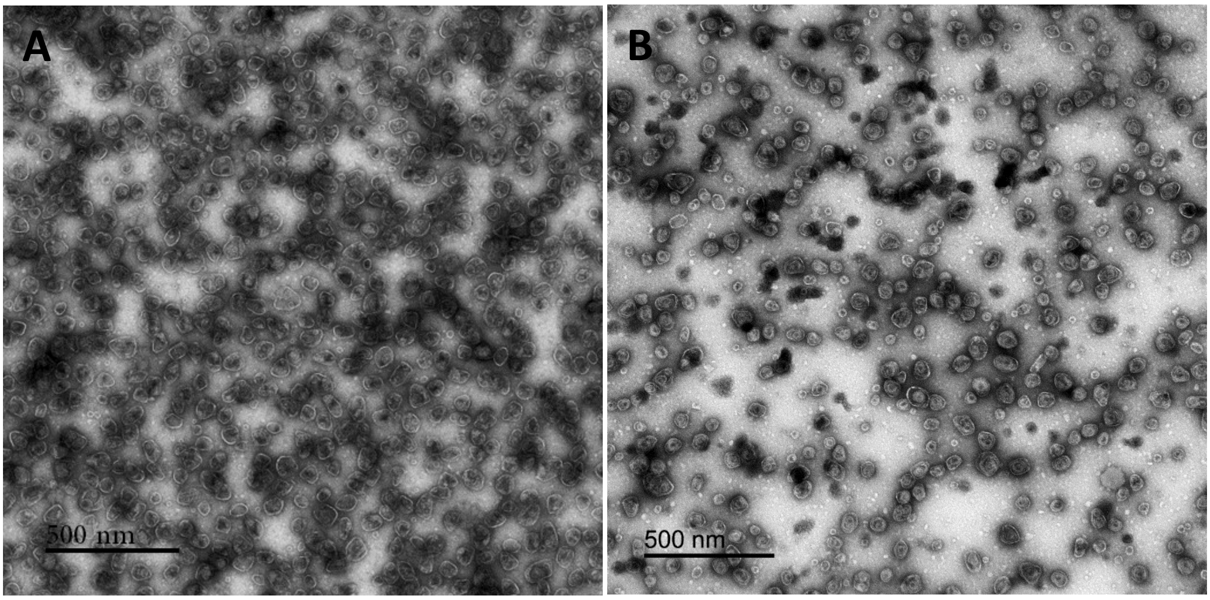

Supplement: MA-004-D3MA00071K-s001 [file MA-004-D3MA00071K-s001.pdf]
